# #BlackLivesMatter

**DOI:** 10.1093/jamiaopen/ooaa032

**Published:** 2020-07-26

**Authors:** Indra Neil Sarkar

**Affiliations:** o1 Rhode Island Quality Institute, 50 Holden Street, Suite 300, Providence, Rhode Island 02908, USA; o2 Center for Biomedical Informatics, Brown University, Box G-R, Providence, Rhode Island 02912, USA

*JAMIA Open* stands firm with colleagues across AMIA and the international biomedical informatics community in opposition to racism. Systemic racism is not acceptable, and yet so much of modern society is hindered by its acceptance. The death of George Floyd on May 25, 2020, was not the first such aggression due to race, and it will, unfortunately, not be the last unless education, understanding, and alliance are achieved by the people of the United States and the world. Protests alone will not bring about change, and we must, together, stand together for equity. 

Oxford University Press (OUP) has been organizing content across its portfolio of publications to encourage discussion pertaining to racial inequality and diversity: https://academic.oup.com/journals/pages/race-and-diversity.

Change can only be brought upon by action. The AMIA Board of Directors has announced the creation of the AMIA Diversity, Equity, and Inclusion Task Force that will chart a path for AMIA.^1^ Diversity, equity, and inclusion are not addressed with simple actions; they are complex challenges that will require innovative solutions. *JAMIA Open* looks forward to receiving guidance on how it can improve our processes and policies to ensure diversity, equity, and inclusion.

As an Editor-In-Chief, my primary responsibility is to you, the readership of *JAMIA Open*, in that I work with a team of Associate Editors and an Editorial Board to select the highest quality research. As should be expected of any journal in biomedicine, our editorial and review processes focus on the science of the work being presented. Still, I am certain there are ways that we can improve our processes to ensure diversity, equity, and inclusion.

Informatics approaches have been shown to be a powerful approach to identify and address health inequity, which is often correlated with a number of social factors (including race). Reports of research in these areas are often limited, and the majority focus on detecting or reducing disparities (as highlighted in the *JAMIA* “Health Informatics and Health Equity: Improving our Reach and Impact” Special Issue^2^). There is a need for more articles to describe approaches for understanding the causes of health inequity. As a gold open access journal, and amongst the first with community-facing abstracts, I encourage submissions on this topic, especially as they relate to understanding the role of the race alongside the myriad social factors that negatively impact health.

I am not naïve in stating that the changes that we make now will have an immediate effect. However, I do believe that the right actions now will result in change for the generations of informaticians that will follow. I know that we can do better.

## Conflict of interest statement

None declared. 
